# Systematic Review and Meta-Analysis of Human Papillomavirus Prevalence and Genotypic Disparities Among Human Immunodeficiency Virus-Positive Women in Africa

**DOI:** 10.3390/jcm14175924

**Published:** 2025-08-22

**Authors:** Yirga Amare, Dahabo Gelgalo, Éva Pozsgai, István Kiss

**Affiliations:** 1 Doctoral School of Medical Sciences, University of Pécs, Szigeti Str. 12, 7624 Pécs, Hungary; 2 Doctoral School of Health Sciences, University of Pécs, Vörösmarty Str. 4, 7621 Pécs, Hungary; 3 Department of Public Health, Medical School, University of Pécs, Szigeti Str. 12, 7624 Pécs, Hungary

**Keywords:** Africa, human immunodeficiency virus, human papillomavirus, prevalence

## Abstract

**Background**: Human papillomavirus (HPV) is the most prevalent sexually transmitted infection with significant health implications, especially for women living with human immunodeficiency virus (HIV). The variability in reported prevalence and genotype distribution of HPV among HIV-positive women across different regions in Africa necessitates a comprehensive and systemic examination. **Methods**: A systematic search was conducted across several databases. A random effect model was used to evaluate study heterogeneity through Q statistics and I^2^ measures. Publication bias was assessed using funnel plots and Egger’s tests. Risk factors for HPV among HIV-positive women were summarized qualitatively. This review was registered with PROSPERO: CRD42024525123. **Result**: Twenty-three studies involving 9954 HIV-positive women were combined to estimate HPV prevalence. The overall prevalence of all HPV types was 49.4% (95% CI: 42.43, 56.38), with evidence of heterogeneity (Q = 520.92, df = 16, I^2^ = 96.93%, *p* < 0.0001). The prevalence of high-risk HPV was 45.26% (95% CI: 31.02, 59.91), showing heterogeneity across studies (Q = 439.18, df = 10, *p* < 0.0001, I^2^ = 97.72%). Low-risk HPV had a prevalence of 24.98% (95% CI: 12.27, 40.41), with variation among studies (Q = 134.39, df = 6, *p* < 0.0001, I^2^ = 95.54%). The most frequent genotypes were 16, 18, 52, 33, and 35. A higher cluster of differentiation 4 (CD4) count is associated with a lower prevalence of HPV. **Conclusions**: The pooled HPV prevalence among HIV-positive women in Africa is lower compared to previous studies, but the slow decline poses challenges to meet the WHO’s goal of eliminating HPV-related cervical cancer by 2030. Therefore, enhanced prevention efforts, including HPV self-sampling, improved vaccination coverage, and early treatment interventions, are essential to meet the goal of eliminating HPV-related cervical cancer.

## 1. Introduction

Globally, the human papillomavirus (HPV) is acknowledged as the most prevalent heterogeneous group of DNA viruses that are sexually transmitted [1]. It replicates persistently in dividing cells, hijacks the main cellular process to manipulate the cellular environment, and remains undetected by the immune system [2]. The virus was originally discovered in the early 1930s in the horn of the Cottontail rabbit, and in the 1970s, it was shown to be the primary cause of cervical cancer.

Over 300 HPV genotypes have been studied [1], with over 200 of those genotypes being sexually transmitted [3,4]. The International Agency for Research on Cancer (IARC) classified HPVs as carcinogenic, probably carcinogenic, or not carcinogenic based on their ability to cause cancer [3]. As per this classification, HPV16, HPV18, HPV31, HPV33, HPV35, HPV39, HPV45, HPV51, HPV52, HPV56, HPV58, and HPV59 are classified as carcinogenic (Group 1), while HPV 68 is classified as probably carcinogenic (Group 2A). The remaining HPVs, namely HPV26, HPV30, HPV34, HPV53, HPV66, HPV67, HPV69, HPV70, HPV73, HPV82, HPV85, and HPV97, are linked to infrequent cases of cervical cancer and are deemed possibly carcinogenic to humans (Group 2B) [1,5]. Certain HPV genotypes are associated with the development of cancer, while genotypes 6 and 11 cause benign, proliferative lesions in the skin and mucosa [2].

Until the immune system weakens, most HPV infections in both sexes are asymptomatic. However, they can cause cervical cancer; anogenital warts; and penile, anal, and oropharyngeal cancers, particularly those linked to HPV16 [2,6]. Precancerous cervical lesions and persistent cervical infections that result in dysplastic changes and, ultimately, cervical cancer require HPV infection of the cervix and integration of the viral DNA into the genome of the host cell [1].

Gardasil^®^, a quadrivalent HPV vaccine, has been available recently to protect against HPV genotypes 6, 11, 16, and 18. As an alternative, the US Food and Drug Administration (FDA) has approved and licensed the use of two HPV vaccines: Cervarix™, a bivalent vaccine that includes genotypes 16 and 18, and Gardasil 9, a nanovivalent vaccine that includes HPV genotypes 6, 11, 16, 18, 31, 33, 45, 52, and 58 [3,7].

The interaction of HPV and HIV presents a compounded health challenge, especially in Africa, where the burden of both infections is notably high. HIV-positive women are more vulnerable to persistent HPV infections and an increased risk of developing HPV-related malignancies [1]. More than 90% of cases of cervical cancer have an HPV diagnosis, making it the most common pathogen in females that causes cancer. In addition, mucocutaneous and anogenital warts as well as genital and oropharyngeal cancers are all brought on by HPV. The incidence and prevalence of these medical conditions are significantly influenced by various factors, including HPV genotype, cluster of differentiation 4 (CD4) count, anatomic location sampled, regional conditions, and study population [7]. Compared to women without HIV, women living with HIV have a 5–9-fold higher risk of developing HPV-related cervical cancer. While chronic HIV infection is linked to a high prevalence of diverse HR-HPV-type infections, persistent HPV infection, precancerous lesions, and rapid disease progression, including cervical cancer and newly acquired HIV infection, increases the risk for high-risk HR-HPV infection [8].

Vaccine scarcity and hesitancy imposed dual challenges in achieving equitable vaccination coverage, particularly in low- and middle-income countries. Supply limitations, and even when available, uptake is hindered by widespread hesitancy rooted in misinformation and distrust [9]. Vaccine hesitancy is a challenge and multifactorial in developing nations [10]. Addressing both scarcity and hesitancy requires a well-coordinated global response that strengthens the supply side, ensures fair distribution, implements culturally appropriate education, and engages the community. 

The World Health Organization (WHO) launched the “90-70-90” global program to be implemented by its member countries to significantly reduce the incidence and mortality of cervical cancer caused by HR-HPV infection. However, the global immunization rate remained at 15% of the planned achievement [11].

Despite the availability of effective vaccines and screening tools, a significant knowledge gap exists in limited-resource and underserved areas of developing countries [11,12]. Many people remain unaware of the link between HPV and non-cervical cancer, and misconceptions about vaccine safety and ineligibility for the vaccine continue to hinder uptake [12,13]. Even among healthcare professionals and students, a knowledge gap persists regarding the HPV route of transmission, and the recommended vaccination schedule hinders them from providing effective women’s education and vaccine advocacy [14]. Co-infections with other pathogens, subpar healthcare facilities, high costs of treatment, and unfavourable living conditions all contribute to high prevalence rates [5].

Human papillomavirus remains the most prevalent sexually transmitted infection globally, with amplified consequences for HIV-positive women. Although Africa bears a disproportionate dual burden of HPV and HIV, most existing studies focus on isolated countries or subregions, often providing fragmented insights. On the other hand, the absence of a comprehensive continental perspective has limited the formation of a unified and effective prevention strategy tailored to the African context. Moreover, few review studies have integrated recent data across all African regions, intending to change HPV vaccination and ART coverage. This study addresses the critical gap by synthesizing findings across North, East, West, Central, and Southern Africa to generate up-to-date pooled prevalence estimates, highlight HPV genotype disparity, and summarize factors associated with HPV infection among HIV-positive women in Africa, helping improve screening, vaccination, treatment, and public health strategies, ultimately reducing the burden of HPV complications.

### Objective

The primary objectives of this study were to systematically analyze the pooled prevalence and genotype disparities of Human Papillomavirus (HPV) among HIV-positive women in Africa, providing a comprehensive estimate and identifying the most frequent HPV genotypes, while the secondary objectives were to examine the association between HIV-related factors and HPV prevalence, assess heterogeneity across studies, and highlight regional variations in HPV prevalence that could impact cervical cancer prevention efforts.

## 2. Materials and Methods

### 2.1. Search Strategy

Among HIV-positive women in Africa, the pooled prevalence and HPV genotype disparities were examined through a systematic review and meta-analysis of published articles. The initial search was carried out to prevent asking the same questions twice and to ensure that a current meta-analysis incorporating North African data had not already been carried out on the subject. We searched Google Scholar (Google LLC, Mountain View, CA, USA), Embase (Elsevier B.V., Amsterdam, The Netherlands), Scopus (Elsevier B.V., Amsterdam, The Netherlands), PubMed (National Center for Biotechnology Information, Bethesda, MD, USA), and the Ovid database from Medline (Ovid Technologies, Wolters Kluwer, New York, NY, USA). Boolean operators [AND, OR] and truncations (*) combined the previously identified search hits to perform database searching. The Preferred Reporting Items for Systematic Reviews and Meta-Analyses (PRISMA framework) standard was adhered to during the review process. During electronic database searching, keywords found in the titles or abstracts of related studies, medical subject headings (MeSH and Emtree terms), and an analysis of the search tactics employed in related or prior systematic reviews were further refined and applied.

In addition, a bibliographic search of papers meeting the inclusion criteria was added to the search strategy. Following the search, every study record found in each database was collected into a reference manager.

### 2.2. Selection Criteria

Studies qualify if the following were included in their report: (1) original English-language articles or abstracts showing the prevalence of HPV infection among HIV-positive women; (2) HPV prevalence was examined using DNA from endometrial and cervical specimens; (3) cohort and cross-sectional research.

Studies were considered ineligible if they met the following requirements: (1) research estimating or displaying the frequency of HPV infection in HIV-negative women; (2) the frequency of HPV infection in males and transgender women; (3) serologic assays like ELISA that quantify HPV antibodies; (4) languages other than English are used to write articles; (5) case reports, posters, and conference papers.

### 2.3. Data Extraction and Quality Assessment

This paper’s two authors independently chose pertinent publications before screening titles and abstracts. We continued reading the abstracts and titles of the articles to extract pertinent data. An Excel spreadsheet was created with a data abstraction form to collect data from the publications. These data included the author’s name, the year the work was published, the sample size, the mean or median age, the study design, the name of the countries, the prevalence of HPV among HIV-positive women, the detection method used, and the HPV genotype. A quality assessment tool was used to evaluate the methodological quality of cross-sectional and cohort studies [15]. The authors’ disagreements about the quality of the approaches and paper selection were resolved through discussion (YW and DG) and consulting the other author (KI). Research exhibiting a quality assessment average score of 50% or higher was included in the final analysis. Attempts were made to contact the corresponding authors of the original articles to access information about incomplete and ambiguous data.

### 2.4. Study Inclusion and Exclusion

The included studies were cross-sectional and cohort studies carried out on the African continent, written in English, and available online between 12 August 2019 and 11 May 2024. Additionally, only publications detailing the prevalence of HPV among women living with HIV were eligible for inclusion. Excluded from consideration were studies carried out on HIV-negative women, studies conducted outside of Africa, and studies that did not provide information about study design or measured outcomes.

### 2.5. Risk of Bias Assessment and Its Implications

**Figure 1:**
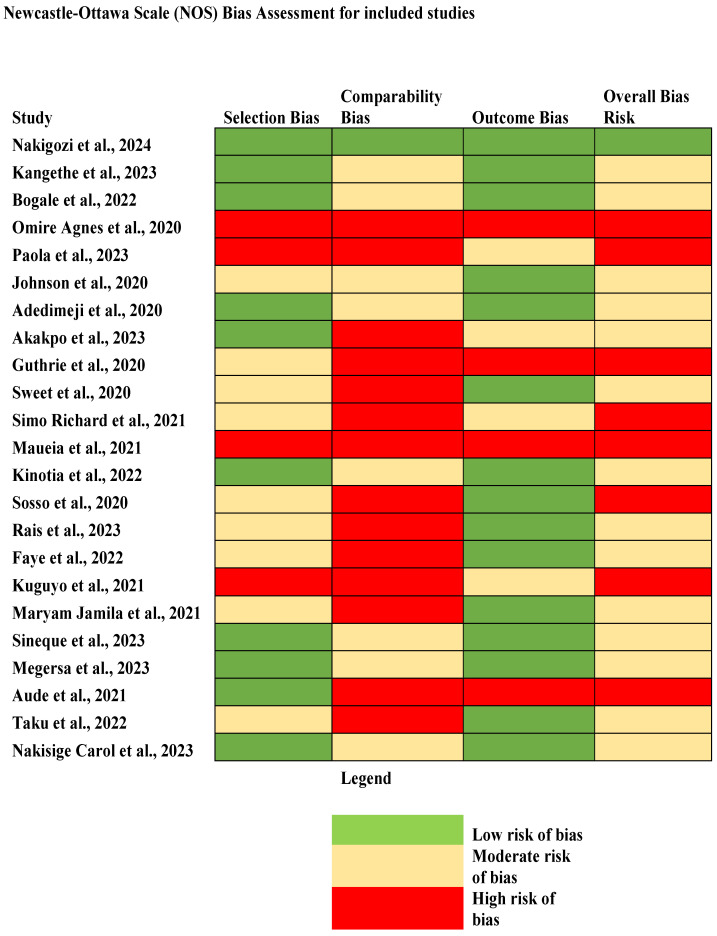
Risk of bias assessment for the included studies [16,17,18,19,20,21,22,23,24,25,26,27,28,29,30,31,32,33,34,35,36,37,38].

The risk of bias assessment for the 23 studies evaluating risk of bias indicates that selection and outcome domains were generally of low concern; however, the comparability domain showed shortcomings, as many studies lacked sufficient control for confounding factors such as age or ART status. Owing to these facts, the majority of the studies were judged to have an overall risk of bias, with a smaller proportion of studies demonstrating a high risk of bias due to many limitations. This suggests that although most studies provide crucial data, the certainty of their findings may be constrained, especially the pooled estimates of HPV, which have to be interpreted with caution in light of these methodological weaknesses (Figure 1).

### 2.6. Data Analysis

The analysis was conducted using Med Calc statistical and Comprehensive Meta-analysis version 4 (CMA) software. The heterogeneity of the studies was evaluated using the I^2^ test and the Q test (*p* < 0.1) based on the assumption of the random effect model. The effect of covariate (publication year) on the heterogeneity of effect sizes was investigated using meta-regression, and subgroup analysis was used to estimate effect sizes within a group and investigate a possible source of heterogeneity in the combined effect. The 95% confidence interval, the pooled effect size comparable to prevalence, and the weight of each study were displayed on a forest plot. The specific genotypes of HPV among HIV-positive women were presented graphically. By calculating the significance of Egger’s and Begg’s tests and examining the symmetry of the funnel plot graph, publication bias was evaluated. This study also included qualitative data in an attempt to define variables that are associated with HPV infection in women living with HIV.

The protocol for this systematic review and meta-analysis was registered in PROSPERO (International Prospective Register of Systematic Reviews) under registration number CRD42024525123 and is available at https://www.crd.york.ac.uk/PROSPERO/view/CRD42024525123 (accessed on 25 April 2024).

## 3. Results

### 3.1. Study Characteristics

**Figure 2:**
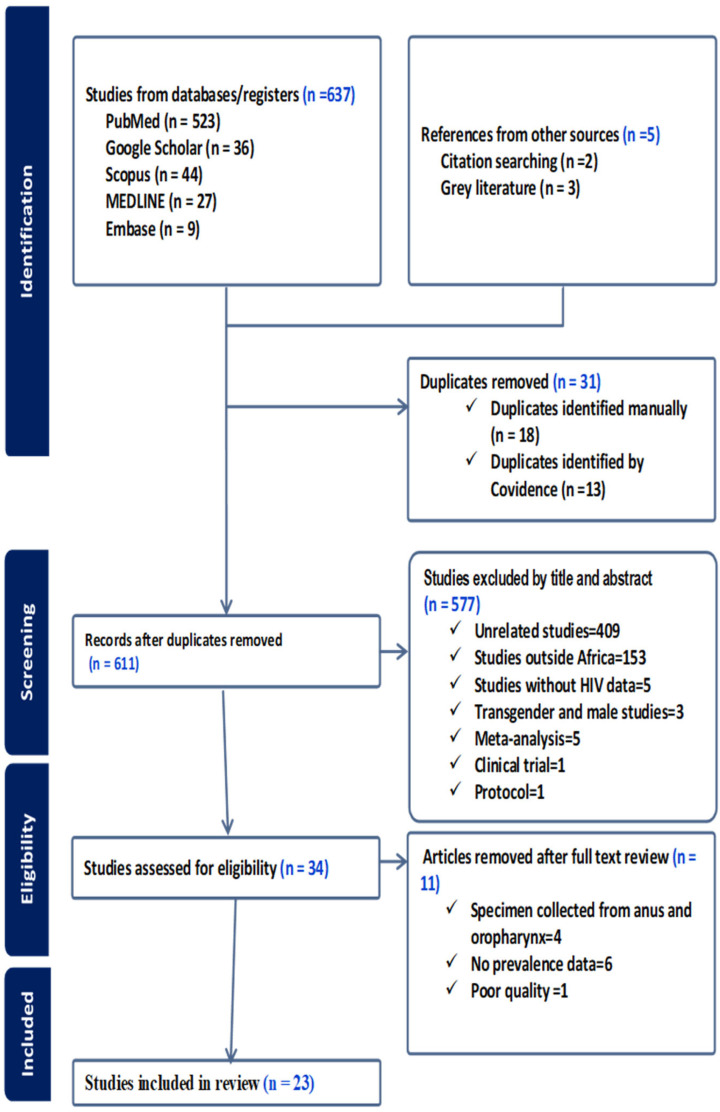
A PRISMA 2020 flowchart [39], showing the steps for selecting eligible studies.

We assessed 642 studies retrieved from different databases using search strategies. Twenty-three (23) cross-sectional and cohort African studies conducted at various study settings between 12 June 2019 and May 2024 were included for analysis. All the procedures we followed to select eligible studies are depicted below (Figure 2).

These studies are reported from Ghana [16], Ethiopia [17,18], Kenya [19,20,21,22,23], South Africa [24,25], Congo [26], Uganda [27,28], Algeria [29], Senegal [30], Zimbabwe [31], Nigeria [32], Mozambique [33,34], Mali [35], and Cameroon [36,37,38].

The total sample size was 9954, with a minimum and maximum individual study sample size of 29 and 5856, respectively. Each study expressed the age of participants using either the median or mean as a measure of central tendency, along with standard deviation, interquartile range, and range as measures of dispersion. Among 23 studies for which quality assessment was performed, 17 studies reported the prevalence of all types of HPV, and 11 and 7 studies reported the prevalence of high-risk (Hr) and low-risk (Lr) HPV, respectively. The maximum and minimum sample sizes are 5856 and 29. The quality assessment results of the included studies vary between a maximum of 95% and a minimum score of 56.5%. Table 1.

### 3.2. Pooled Prevalence of HPV

#### 3.2.1. Pooled Prevalence of All Types of Human Papillomavirus (HPV) Infection

**Figure 3:**
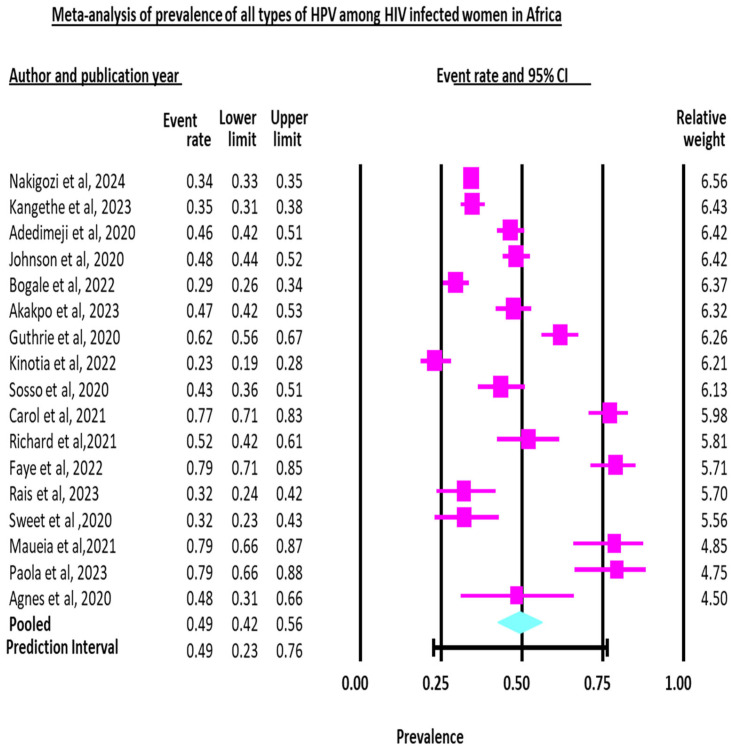
Forest plot showing the pooled prevalence of all types of HPV infection among HIV-positive women in Africa. ES (effect size) = prevalence, CI (confidence interval) is represented by dash (-), diamond represents pooled prevalence (ES), and boxes show individual studies’ ES. The dash indicates the length of the CI, and the size of the box shows the weight based on the random effect model [16,17,19,20,21,22,23,24,26,27,28,29,30,33,36,37,38].

We pooled data from 23 studies comprising 9954 HIV-positive women to estimate the pooled prevalence of all types of HPV infection. The total pooled prevalence of all types of HPV infection among HIV-infected women is 0.494 with a 95% confidence interval of 0.42 to 0.56, and the true effect size in 95% of all comparable populations falls in the interval 0.23 to 0.77. The heterogeneity among the studies is Q = 520.92, with df = 16, I^2^ = 96.93%, and *p* < 0.0001. Close to 97% of the observed variance (I^2^) reflects the variance in the true effect rather than the sampling error. Conversely, 3% of the variance in effect is due to sampling error (Table 2 and Figure 3). Heterogeneity is high, which suggests significant differences between studies, which means a single summary statistic might not be representative of all populations.

#### 3.2.2. Pooled Prevalence of High-Risk Human Papillomavirus Infection

**Figure 4:**
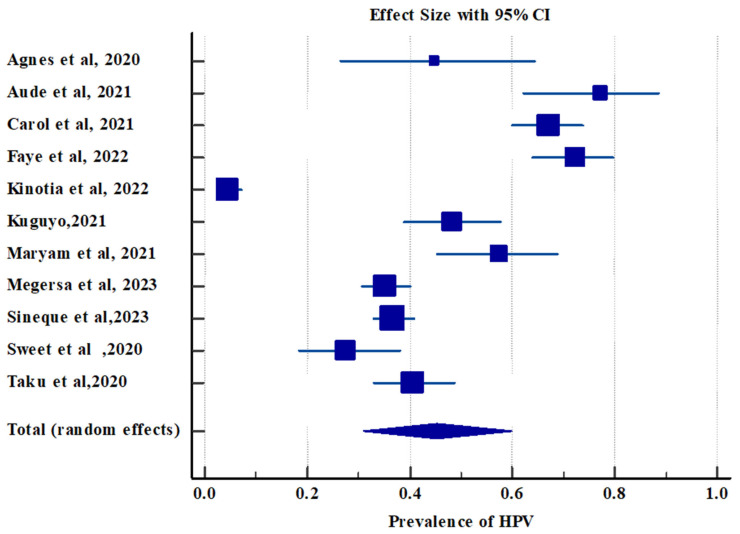
Forest plot showing the pooled prevalence of Hr HPV infection among HIV-positive women in Africa. ES (effect size) = prevalence, CI (confidence interval) is represented by dash (-), diamond represents pooled prevalence (ES), and boxes show individual studies’ ES. The dash indicates the length of CI, and the size of the box shows the weight based on the random effect model [18,21,22,23,25,28,30,31,32,34,35].

We pooled the data of 2124 HIV-positive women from 11 studies to analyze the pooled prevalence of high-risk (Hr) HPV. The pooled prevalence of Hr HPV infection among HIV-positive women was 45.26 (95% CI; 31.027, 59.914) with evidence of heterogeneity across the studies (Q = 439.1812, DF = 10, significance level = *p* < 0.0001, I^2^ = 97.72%, and 95% CI for I^2^ = (96.95, 98.30)) (Table 3 and Figure 4).

#### 3.2.3. Pooled Prevalence of Low-Risk Human Papillomavirus Infection

**Figure 5:**
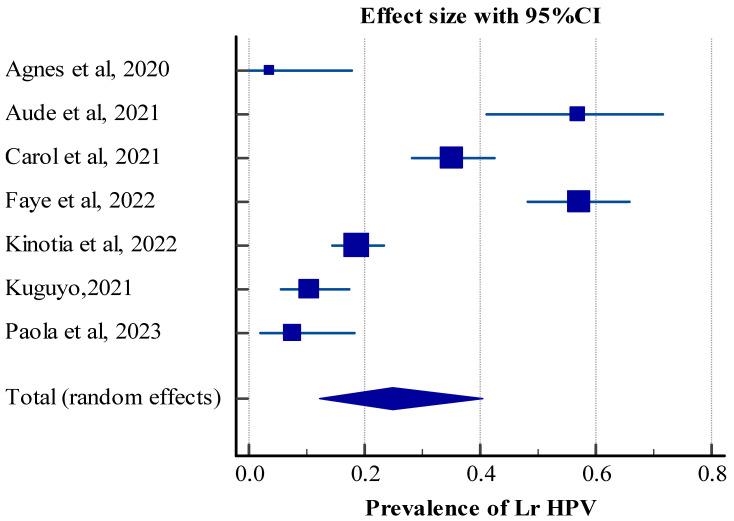
Forest plot showing the pooled prevalence of Lr HPV infection among HIV-positive women in Africa. ES (effect size) = prevalence, CI (confidence interval) is represented by dash (-), diamond represents pooled prevalence (ES), and boxes show individual studies’ ES. The size of the dash indicates the length of CI and the size of the box shows the weight based on the random effect [21,23,26,28,30,31,35].

Data from seven studies containing a total of 880 participants were pooled to estimate the prevalence of low-risk human papillomavirus among HIV-infected women. The result showed that the pooled prevalence of Lr HPV infection is 24.98 (95% CI: 12.27, 40.41). The test of heterogeneity indicated the existence of heterogeneity among the studies (Q = 134.39, DF = 6, significance level= *p* < 0.0001, I^2^ = 95.54%, and 95% CI for I^2^ = (92.89 to 97.20)) (Table 4 and Figure 5).

### 3.3. Sensitivity Analysis

**Figure 6:**
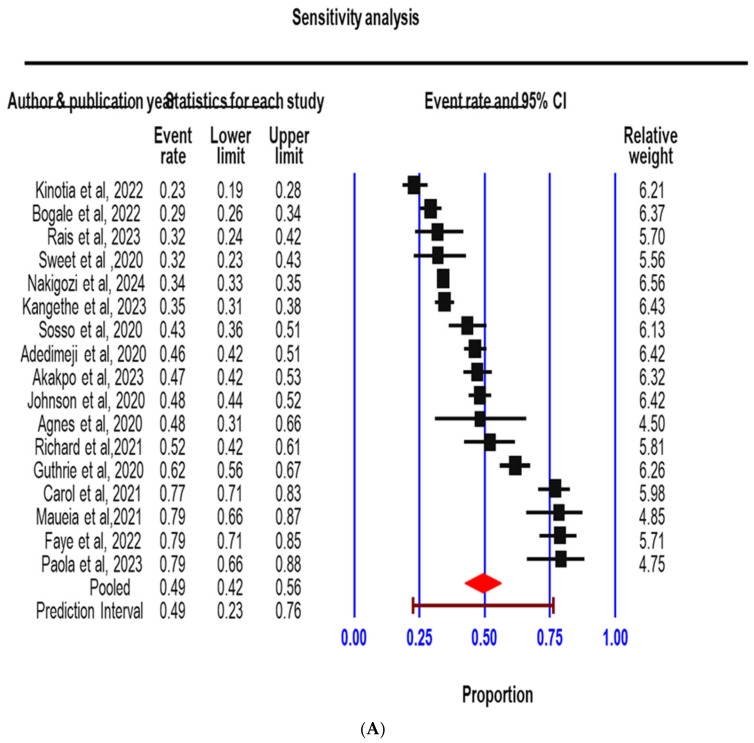

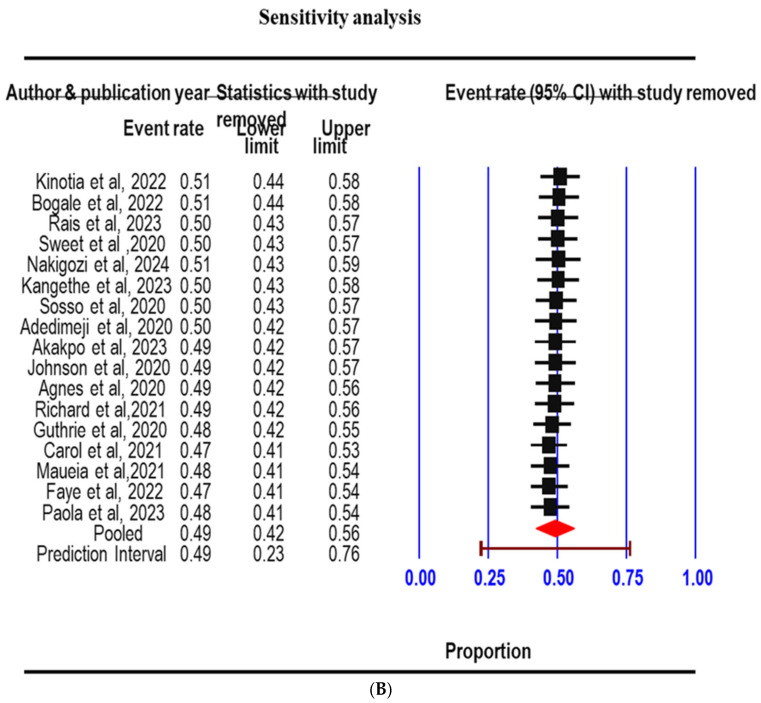
(**A**): A forest plot of event rate sequenced from the lowest to the highest. (**B**): A forest plot with one study removed [16,17,19,20,21,22,23,24,26,27,28,29,30,33,36,37,38].

Relative weight for each study was computed, and no study obtained a value greater than 6.56. This implies there was no one study dominating this analysis. To ensure the basic conclusion was not dependent on one study, we sorted the event rates from low to highest (Figure 6A). After sorting, we conducted a sensitivity analysis with one study removed (Figure 6B). The result indicated that the average effect size for the remaining 16 studies falls between 0.48 and 0.51. That means the average effect size did not shift meaningfully when any one study was removed.

### 3.4. Subgroup Analysis

**Figure 7:**
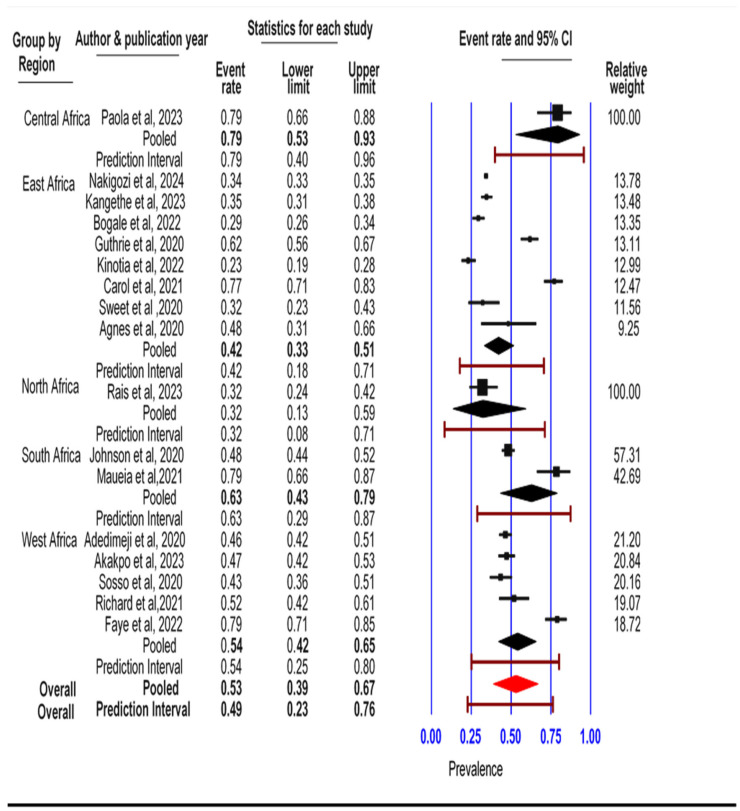
Subgroup analysis of the prevalence of HPV among HIV-positive women by regions in Africa. CI (confidence interval) is represented by a dash (-), a diamond represents pooled prevalence (ES), and boxes show individual studies’ ES. The size of the dash indicates the length of CI, and the size of the box shows the weight based on the random effect [16,17,19,20,21,22,23,24,26,27,28,29,30,33,36,37,38].

Sub-group analysis based on the random effect assumption was carried out for five regions of Africa, namely North, South, Central, East, and West Africa, where primary studies originated for inclusion. The result showed significant heterogeneity within and between the groups. The pooled prevalence of HPV among HIV-infected women in the East Africa region was 0.42 (95% CI: 0.33, 0.51) with a prediction interval between 0.18 and 0.71, I^2^ = 96.8, and *p*-value = 0.00. The pooled prevalence of HPV in the West Africa region was 0.54 (95% CI: 0.42, 0.65), prediction interval = 0.201–0.842, I^2^ = 91.2, and *p*-value = 0.00. The South Africa region had a 0.63 (95% CI: 0.43, 0.79) pooled prevalence of HPV infection and a measure of heterogeneity I^2^ = 94%, *p*-value < 0.001, and tau square = 0.882 (Figure 7).

### 3.5. Meta-Regression

The effect of publication year on the heterogeneity of effect sizes between studies was examined using a meta-regression model. Using the criterion alpha 0.05, we retain the null hypothesis that no significant relationship exists between publication year and effect size (*p* = 0.337). We conducted model tests to assess whether the predictor variable contributes to variation in effect size and to evaluate the overall fit of the model to the data. Testing of the model using a criterion alpha of 0.1 yielded a *p*-value of 0.337. Therefore, since *p*-value is greater than 0.1, we retain the null hypothesis that publication year does not explain any of the variations in effect. The goodness-of-fit test showed a *p*-value of 0.00. Therefore, based on the criterion alpha of 0.1, we rejected the null hypothesis that publication year does not explain all of the variations in effect sizes. By combining the two proven hypotheses, we concluded that publication year explains only some variation in effect size. The R^2^ analogue is 0.02, indicating that the covariate or publication year explains only 2% of the variation in true effects (Table 5).

**Figure 8:**
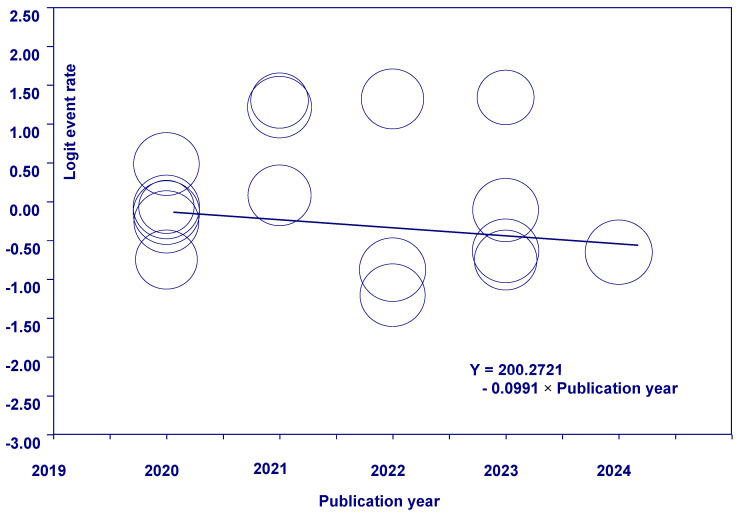
Regression of logit event rate on publication year.

The graph shows that for each additional year of publication, the effect size decreases by 0.0991 logit units. For studies where the publication year is 2020, the event rate in the logit unit was 0.0901 (Y = 200.2721 − 0.0991 × 2020) or close to a 52% event rate, and for studies where the publication year is 2024, the event rate in the logit unit was −0.3063, which is roughly a 42% prevalence. So, as the publication year increases by approximately 5 years (2020–2024), the effect size decreases roughly by −2.197 logit units or 10% prevalence (Figure 8).

### 3.6. Publication Bias

**Figure 9:**
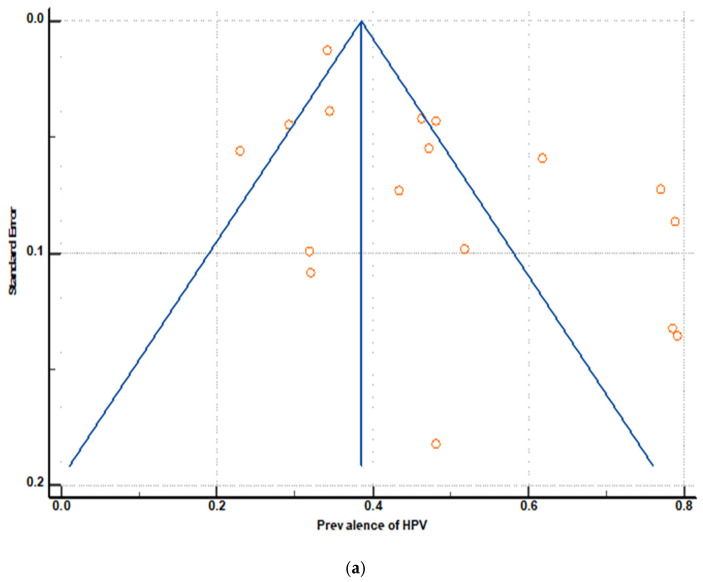

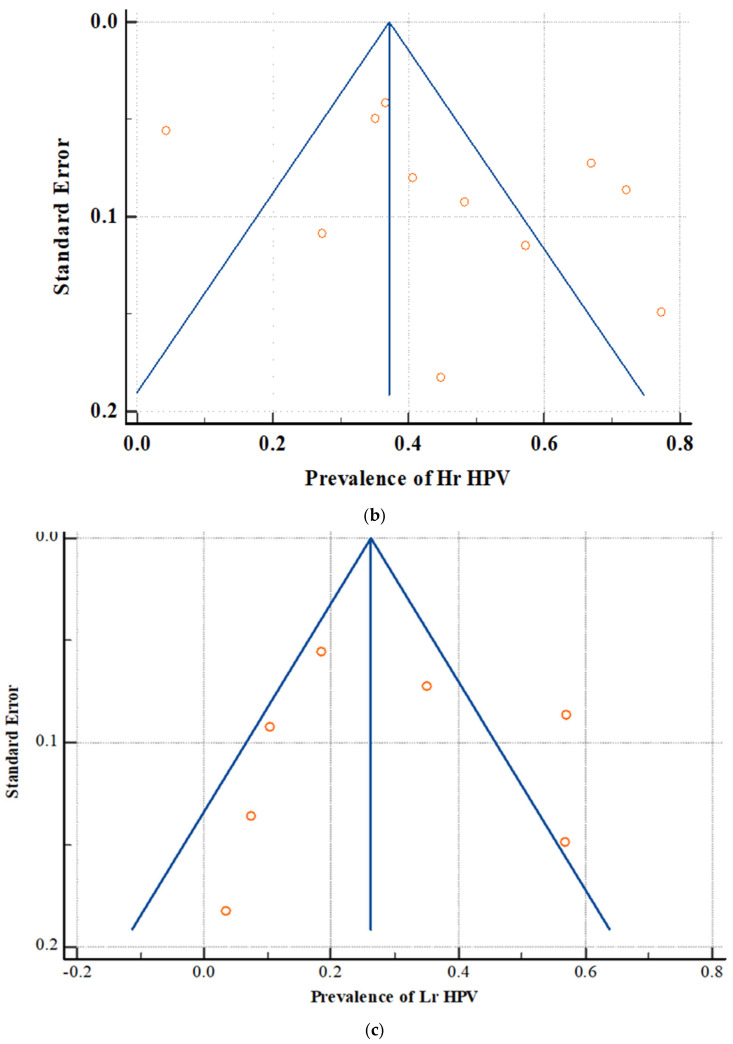
(**a**): Funnel plot for assessment of publication bias among 17 effect size estimates of all forms of HPV. (**b**): Funnel plot for assessment of publication bias among 11 effect size estimates of Hr HPV. (**c**): Funnel plot for assessment of publication bias among 7 effect size estimates of Lr HPV.

Visual inspection of the funnel plot graph, along with Egger’s and Begg’s tests, was used to determine publication bias in the pooled prevalence data for all genotypes, high-risk and low-risk HPV. The funnel plots of 17 studies for all genotypes of HPV indicate asymmetric distribution, implying publication bias (Figure 9a), and this is further supported by the Egger’s test (effect estimate: 5.05 (95% CI; 1.28–8.82), *p* = 0.01 as the intercept is different from 0, and *p* is less than 0.05) (Table 6). The overall assessment suggests possible publication bias, meaning that studies with negative or non-significant results may be missing because they were not published, leading to overestimation or underestimation of the true effect size.

The funnel plots of 11 studies for high-risk HPV showed an asymmetric distribution, implying publication bias (Figure 9b). However, this is not evidenced by both Egger’s test (Effect estimate: 6.4976 (95% CI; −4.53 to 17.52), *p* = 0.21 and Begg’s test (Z = 0.1636, *p* = 0.4835), in which case *p* is not significant).

Symmetrical distribution of effect size estimates was observed for seven studies analyzed for low-risk HPV among HIV-positive women (Figure 9c), and this is further supported by Egger’s test and Begg’s test, in which case the *p* value is greater than 0.05.

### 3.7. HPV Genotype Disparities Among HIV-Positive Women

**Figure 10:**
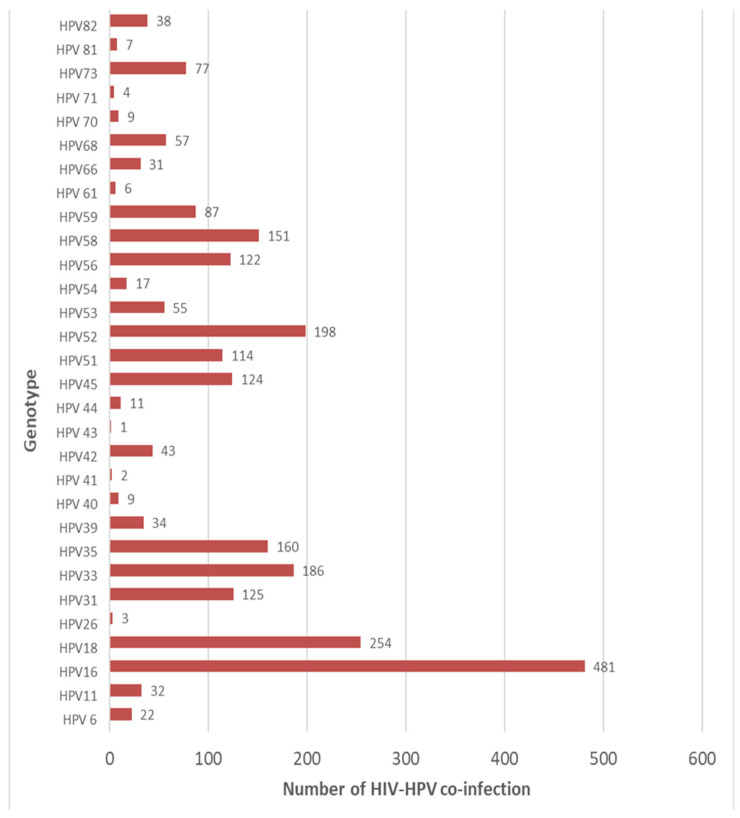
Distribution of HPV-specific genotypes among HIV-positive women in Africa.

Human papillomavirus genotypes 16, 18, 52, 33, 35, 58, 31, 45, 56, and 51, consecutively, are the 10 top frequently reported genotypes among HIV-positive women in Africa. However, genotypes 43, 41, and 26 are less common in Africa (Figure 10).

### 3.8. Factors Associated with HPV Infection

A higher CD4 count is associated with a lower prevalence of HPV [18,21,28,36,37]. Self-collected specimens detect more oncogenic HPV than specimens collected by healthcare providers [17,37]. There is also an association between younger age (age less than 30) and HPV infection among HIV-infected women. Women with high viral load (1000 copies/mL) [18,37] and who have never been pregnant had a higher chance of being infected with HPV [16]. Abnormal cytology (dysplasia) is associated with multiple infections with HPV among HIV-positive women [23,37]. (Table 7).

## 4. Discussion

This systematic review and meta-analysis were conducted using representative African studies to provide an up-to-date summary of the pooled and genotype-specific prevalence of HPV infection, as well as associated factors, among HIV-infected women in the African region.

According to this study, the pooled prevalence of all genotypes and high-risk and low-risk human papillomavirus infection among HIV-positive women was 49.4%, 45.2%, and 24.9%, respectively. This is lower than the prevalence reported in a study conducted in sub-Saharan countries in 2020, which reported prevalence rates of 63%, 51%, and 28% [1]. Similarly, our study found that the prevalence of high-risk HPV was lower than reported in a systematic review and meta-analysis conducted in Kenya in 2016, which reported a prevalence rate of 64% [40]. This decline in the prevalence might be attributed to improvements in HPV vaccination and ART (antiretroviral therapy) coverage.

The prevalence of all types of HPV varies from the lowest in Algeria (32.1%) [29] to the highest in Congo (79.2%) [26]. This difference could be due to differences in sexual behavior, WHO stage of HIV infection, CD4 count at the time of HPV testing, vaccination coverage, screening habits, and socio-economic factors. As the HIV stage progresses from WHO stage I to stage IV, immunity or CD4 count tends to decrease, resulting in increased HPV acquisition, reduced viral clearance, and higher HPV reactivation [29]. This is especially true in HIV-infected women who have either not initiated antiretroviral therapy (ART) or have poor adherence to ART.

In agreement with previous studies conducted in 2020 [1] and 2017 [41], our review indicated HPV genotypes HPV 16, 18, and 52 are the most frequently reported genotypes in Africa. These specific genotypes in females are still dominant despite the introduction and availability of the nine-valent vaccine, which can be given as early as 9 years of age. This antigen is protective against HPV types 6, 11, 16, 18, 31, 33, 45, 52, and 58. Although HPV vaccination uptake in resource-limited regions like Africa has seen some positive changes, it is not as expected, with the second dose performing even worse [42]. A higher CD4 count is associated with a low prevalence of HPV infection, which is a similar report to previous studies [1]. This association may be partly explained by the fact that with a high CD4 count, viral clearance increases, and latent HPV would not be apparent and test positive in HIV-infected women. In agreement with a recent study conducted in Nigeria [43], our review revealed that oncogenic HPVs are detected more in self-collected specimens than in specimens collected by healthcare providers.

Even though the decline is not significant (*p* = 0.337), this study suggests that for every one-year increment in the publication year, there was a 0.1 unit decline in the prevalence of HPV infection, which has a similar declining trend to a previous study conducted in sub-Saharan Africa [1]. If the current sluggish decline trend persists, it is probably impossible for Africa to catch the World Health Organization’s (WHO) call for eliminating HPV-related cervical cancer by 2030.

The subgroup analysis in this study showed the highest pooled prevalence of HPV among HIV-positive women in Central Africa (79%), followed by Southern Africa (63%), and the West Africa region (54%). East Africa and North Africa shared 42% and 32% pooled prevalence of HPV, respectively. These disparities in the pooled prevalence of HPV across the regions and within the Southern, West, and East Africa regions may be attributed to the availability of test service and quality of collected data, screening method used and selection of samples (provider-collected sample vs. self-sample or vaginal vs. cervical specimen).

In this study, heterogeneity is high, suggesting significant differences between studies, which means a single summary statistic might not be representative of all populations. Potential clinical sources for this variation can be the study place, such as hospital-based versus community-based studies, which affects sample selection and disease prevalence. Population characteristics like sexual activity and immunosuppression influence HPV prevalence. However, this evidence is suggestive rather than perfect as the studies included in this study are limited in number plus have small sample sizes. Similarly, studies that did not show heterogeneity must not be taken as perfect evidence.

### Limitations of the Study

We included only study articles written in English. This could be one reason for the observed and computed evidence of publication bias and possibly the heterogeneity of estimates of effect sizes. The small sample size in some included studies is another compelling and overarching limitation of this study. Furthermore, this review did not bridge the existing knowledge gap in HPV prevention.

## 5. Conclusions

The limitations of this study reflect the opportunities for future research and practice. Despite these limitations, the present systematic review and meta-analysis provide a detailed and up-to-date summary of the burden and genotypic disparities of HPV among HIV-positive women in Africa. Our analysis indicated a lower pooled prevalence of HPV infection compared to previous studies conducted in Africa. This reduction in the prevalence and consistent decline over the past 5 years can be seen as a promising indicator that Africa is on track for the elimination of cervical cancer in the long run. Disparities in the effect size estimates among the countries and sub-regions must be addressed. Good practices from countries with low prevalence should be shared with countries with a high prevalence of HPV infection. Programs focused on the prevention of HPV infection and HIV should be further strengthened with resources like the HPV vaccine. Efforts to accelerate the elimination of HPV infection and vaccine hesitancy should be addressed to shorten the period required for eliminating HPV and cervical cancer among this vulnerable population. To achieve the World Health Organization’s ambitious target of eliminating cervical cancer, it is crucial to strengthen preventive measures. This includes expanding access to HPV self-sampling, ensuring broader and more effective vaccination coverage, and implementing timely treatment interventions. By improving these key areas, public health efforts can significantly reduce the incidence of HPV-related cervical cancer and move closer to the goal of eradication.

## Figures and Tables

**Table 1:** Characteristics and quality of included studies reporting HPV cases among HIV-positive women in Africa, 2024.

Author & Year	Study Location	Study Design	Study Setting	Age of Study Subjects	Sample Size	Total Event	Hr HPV	Lr HPV	Quality Assessment %
Adedimeji et al., 2020 [37]	Cameroon	Cross-sectional	HIV treatment center	Median = 42 Range = 25–59	560	260	-	-	84
Akakpo et al., 2023 [16]	Ghana	Cross-sectional	Hospital	Mean = 47.2 std = 10	330	156	-	-	74.5
Bogale et al., 2022 [17]	Ethiopia	Cross-sectional	Hospital	-	497	146	-	-	85
Guthrie et al., 2020 [19]	Kenya	Cross-sectional	-	-	283	175	-	-	69.5
Johnson et al., 2020 [24]	S. Africa	Cross-sectional	University	Range = 30–65	535	258	-	-	77.5
Kangethe et al., 2023 [20]	Kenya	Cross-sectional	Hospital	Mean = 42.8 std = 8.7	647	224	-	-	88.5
Paola et al., 2023 [26]	Congo	Cross-sectional	Hospital	Mean = 53 Std = 18.6	53	42	-	4	65
Sweet et al., 2020 [22]	Kenya	Cross-sectional	Clinic	Median = 32	84	27	23	-	68.5
Nakigozi et al., 2024 [27]	Uganda	Cross-sectional	Clinic	Range = 25–49	5856	2006	-	-	95
Richard, Simo et al., 2021 [38]	Cameroon	Cross-sectional	Health center	Range = 20–84	102	53	-	-	66.5
Maueia et al., 2021 [33]	Mozambique	Cross-sectional	-	Range = 14–45	56	44	-	-	64.5
Kinotia et al., 2022 [23]	Kenya	Cross-sectional	Hospital	Mean = 34.3 std = 10.4	317	73	14	59	79
Sosso et al., 2020 [36]	Cameroon	Cross-sectional	Hospital	Mean = 37 std = 3	184	80	-	-	68
Omire, Agnes et al., 2020 [21]	Kenya	Cross-sectional	Hospital	Mean = 40.7 std = 6.6	29	14	13	1	56.5
Rais et al., 2023 [29]	Algeria	Cross-sectional	-	-	100	32	-	-	70
Faye et al., 2022 [30]	Senegal	Cross-sectional	Hospital	≥20	133	105	96	76	71
Kuguyo et al., 2021 [31]	Zimbabwe	Cross-sectional	Hospital	Median = 46 IQR = 41–54	116	-	56	12	53
Jamila, Maryam et al., 2021 [32]	Nigeria	Cross-sectional	Hospital and clinic	Median = 49.5 IQR = 41.4–55.0	75	-	43	-	66
Sineque et al., 2023 [34]	Mozambique	Cross-sectional	Hospital	Median = 43 IQR = 38–47	577	-	212	-	88
Megersa, Tariku et al., 2023 [18]	Ethiopia	Cross-sectional	Hospital	Mean = 34 std = 6	406	-	143	-	75.5
Aude et al., 2021 [35]	Mali	Cross-sectional	Clinic	Median = 40 IQR = 34–44	44	-	34	25	65
Nakisige, Carol et al., 2023 [28]	Uganda	Cohort	Clinic	Median = 34 IQR = 28–40	188	145	126	66	74
Taku et al., 2022 [25]	South Africa	Cross-sectional	Clinic	Median = 46 IQR 38–55	155	-	63	-	73

Legend: Hr = high-risk; Lr = low-risk; - = not reported.

**Table 2:** Single-arm meta-analysis of the prevalence of all types of HPV among HIV-positive women in Africa, 2024.

Study	Sample Size	Prevalence (%)	95% CI	Weight (%) Random
Adedimeji et al., 2020 [37]	560	46.429	42.23 to 50.65	6.28
Agnes et al., 2020 [21]	29	48.276	29.44 to 67.46	4.52
Akakpo et al., 2023 [16]	330	47.273	41.78 to 52.81	6.19
Bogale et al., 2022 [17]	497	29.376	25.40 to 33.59	6.27
Carol et al., 2021 [28]	188	77.128	70.45 to 82.92	6.02
Faye et al., 2022 [30]	133	78.947	71.03 to 85.53	5.87
Guthrie et al., 2020 [19]	283	61.837	55.90 to 67.52	6.15
Johnson et al., 2020 [24]	535	48.224	43.91 to 52.55	6.28
Kangethe et al., 2023 [20]	647	34.621	30.95 to 38.42	6.30
Kinotia et al., 2022 [23]	317	23.028	18.50 to 28.06	6.18
Maueia et al., 2021 [33]	56	78.571	65.56 to 88.40	5.26
Nakigozi et al., 2024 [27]	5856	34.255	33.04 to 35.48	6.41
Paola et al., 2023 [26]	53	79.245	65.89 to 89.15	5.21
Rais et al., 2023 [29]	100	32.000	23.02 to 42.07	5.71
Richard et al., 2021 [38]	102	51.961	41.84 to 61.96	5.72
Sosso et al., 2020 [36]	184	43.478	36.20 to 50.96	6.02
Sweet et al., 2020 [22]	84	32.143	22.36 to 43.22	5.60
**Total (random effects)**	**9954**	**49.405**	**42.43 to 56.38**	**100.00**
**Prediction interval**		**49.4**	**0.23 to 0.77**	
**Test for heterogeneity**				
Q	520.9266			
DF	16			
Significance level	*p* < 0.0001			
I^2^	96.93%			
95% CI for I^2^	96.03 to 97.62			

**Table 3:** Single-arm meta-analysis of the prevalence of Hr HPV infection among HIV-positive women in Africa, 2024.

Study	Sample Size	Proportion (%)	95% CI	Weight (%)	
				Fixed	Random
Agnes et al., 2020 [21]	29	44.82	26.44 to 64.30	1.41	8.32
Aude et al., 2021 [35]	44	77.27	62.15 to 88.52	2.11	8.67
Carol et al., 2021 [28]	188	67.02	59.80 to 73.69	8.85	9.27
Faye et al., 2022 [30]	133	72.18	63.74 to 79.59	6.28	9.19
Kinotia et al., 2022 [23]	317	4.41	2.43 to 7.29	14.89	9.36
Kuguyo, 2021 [31]	116	48.27	38.90 to 57.74	5.48	9.15
Maryam et al., 2021 [32]	75	57.33	45.37 to 68.69	3.56	8.98
Megersa et al., 2023 [18]	406	35.22	30.57 to 40.08	19.06	9.38
Sineque et al., 2023 [34]	577	36.74	32.79 to 40.82	27.07	9.41
Sweet et al., 2020 [22]	84	27.38	18.21 to 38.20	3.98	9.03
Taku et al., 2020 [25]	155	40.64	32.83 to 48.81	7.31	9.23
**Total (random effects)**	**2124**	**45.26**	**31.02 to 59.91**	**100.00**	**100.0**
**Test for heterogeneity**					
Q	439.1812				
DF	10				
Significance level	*p* < 0.0001				
I^2^ (inconsistency)	97.72%				
95% CI for I^2^	96.95 to 98.30				

**Table 4:** Single-arm meta-analysis of prevalence of Lr HPV infection among HIV-positive women in Africa, 2024.

Study	Sample Size	Prevalence (%)	95% CI	Weight (%)	
				Fixed	Random
Agnes et al., 2020 [21]	29	3.44	0.08 to 17.76	3.38	13.00
Aude et al., 2021 [35]	44	56.81	41.03 to 71.65	5.07	13.69
Carol et al., 2021 [28]	188	35.10	28.30 to 42.38	21.31	14.90
Faye et al., 2022 [30]	133	57.14	48.27 to 65.68	15.11	14.74
Kinotia et al., 2022 [23]	317	18.61	14.48 to 23.34	35.85	15.07
Kuguyo, 2021 [31]	116	10.34	5.46 to 17.37	13.19	14.65
Paola et al., 2023 [26]	53	7.54	2.09 to 18.21	6.09	13.94
Total (fixed effects)	880	26.21	23.35 to 29.24	100.0	100.00
Total (random effects)	880	24.98	12.27 to 40.41	100.0	100.00
**Test for heterogeneity**					
Q	134.3977				
DF	6				
Significance level	*p* < 0.0001				
I^2^ (inconsistency)	95.54%				
95% CI for I^2^	92.89 to 97.20				

**Table 5:** Effect of publication year on the heterogeneity of effect sizes.

Main Results for Model, Random Effects (MM), Z-Distribution, Logit Event Rate						
Covariate	Coefficient	Standard Error	95% Lower	95% Upper	Z-Value	*p*-Value
Publication year	−0.0991	0.1034	−0.3017	0.1035	−0.96	0.3378
**Comparison of Model 1 with the null model**						
Q = 0.92, df = 1, *p* = 0.3378						
**Goodness of fit**						
Tau^2^ = 0.2942, Tau = 0.5424, I^2^ = 94.96%, Q = 297.72, df = 15, *p* = 0.0000						
**Total between-study variance (intercept only)**						
Tau^2^ = 0.2983, Tau = 0.5462, I^2^ = 96.36%, Q = 439.85, df = 16, *p* = 0.0000						
**The proportion of total between-study variance explained by Model 1**						
R^2^ analog = 0.02						

**Table 6:** Evidence for publication bias for effect size estimates.

Egger’s Test	All Types of HPV	High-Risk HPV	Low-Risk HPV
Intercept	5.05	6.49	−0.45
95% CI	1.28 to 8.82	−4.53 to 17.52	−14.42 to 13.51
Significance level	*p* = 0.012	*p* = 0.215	*p* = 0.936
Begg’s test			
Kendall’s Tau	0.191	0.163	0.142
Significance level	*p* = 0.284	*p* = 0.483	*p* = 0.652

**Table 7:** Molecular diagnostic methods used and associated factors with HPV infection.

Author & Year	Study Setting	Diagnosis Method	Associated Risk Factor
Adedimeji et al., 2020 [37]	HIV Rx center	Gene Xpert	A higher CD4 count was associated with lower HPV prevalence. HPV testing of self-collected specimens appeared less specific than HPV testing of provider-collected specimens. The prevalence of HPV and HPV16 decreased with increasing age. Self-collected cervicovaginal specimens were more likely to test high-risk HPV positive than the provider-collected cervical specimens.
Akakpo et al., 2023 [16]	Hospital	AmpFire HPV detection system or isothermal PCR assay	Women with HIV viral load ≥ 1000 copies/mL. Women who had 1 or more pregnancies are less likely to have HPV 16 and/or 18 genotypes compared to those who had no pregnancy.
Bogale et al., 2022 [17]	Hospital	Abbott real-time PCR	Oncogenic HPV was higher in self-collected samples than the clinician-collected specimens.
Kangethe et al., 2023 [20]	Hospital	Gene Xpert^®^	WLHIV aged < 25 years had the highest prevalence of high-risk HPV infection. Abnormal cervical cytology and having multiple HR-HPV infections, regardless of ART duration, CD4 count and behavioral factors.
Nakigozi et al., 2024 [27]	ART clinic	HPV DNA gen expert assay and HPV RNA Hologic assay	Compared to women aged 25–35 years, those aged 36–49 had a lower prevalence of all high-risk HPVs.
Richard, Simo et al., 2021 [38]	Health Centre,	HPV DNA genotyping assays and type- pecific PCR	Smoking was associated with a higher prevalence of HPV.
Kinotia et al., 2022 [23]	Referral Hospital	HPV DNA PCR, HPV DNA sequencing	Cervical dysplasia was associated with more mixed low-risk/high-risk HPV genotypes among HIV-infected than HIV-uninfected women.
Sosso et al., 2020 [36]	Hospital	Real-time PCR	The lower the CD4 count, the higher the rate of HPV positivity.
Omire Agnes et al., 2020 [21]	Referral hospital	Roche Linear Array test	Low CD4 T cell count < 200/mm^3^.
Megersa, Tariku et al., 2023 [18]	Hospital	Cobas 4800 HPV Test	End line CD4 count < 200 cells/mm^3^. End-line HIV viral load ≥ 50 copies/mL. More than one lifetime sexual partner was significantly associated with high-risk HPV infections.
Nakisige, Carol et al., 2023 [28]	-	Roche Linear Array test	A lower CD4/8 ratio was significantly associated with measures of high-risk HPV.
Taku et al., 2022 [25]	Clinic	Taxon-directed 16S rRNA gene quantitative PCR (qPCR) assay	HIV-positive women had significantly higher high-risk HPV viral load.

## Data Availability

Data supporting the conclusion of this study are available upon reasonable request of the corresponding author.

## Abbreviations

AIDS	Acquired Immunodeficiency Syndrome
CI	Confidence Interval
DNA	Deoxyribonucleic Acid
HIV	Human Immunodeficiency Virus
HPV	Human Papillomavirus
HR-HPV	High-Risk Human Papillomavirus
I2	I-Squared Statistic Heterogeneity
LR-HPV	Low-Risk Human Papillomavirus
MA	Meta-Analysis
PCR	Polymerase Chain Reaction
PRISMA	Preferred Reporting Items for Systematic Reviews and Meta-Analyses
PROSPERO	Prospective Register of Systematic Reviews
ROB	Risk of Bias
SR	Systematic Review
SRMA	Systematic Review and Meta-Analysis
WLHIV	Women Living with HIV
